# Wnt and BMP signalling direct anterior–posterior differentiation in aggregates of mouse embryonic stem cells

**DOI:** 10.1242/bio.059981

**Published:** 2023-09-13

**Authors:** Atoosa Amel, Alexa Rabeling, Simoné Rossouw, Mubeen Goolam

**Affiliations:** ^1^ Department of Human Biology, University of Cape Town, Cape Town 7925, South Africa; ^2^ UCT Neuroscience Institute, Cape Town, South Africa

**Keywords:** Embryonic morphogenesis, Morphogen gradient, Embryonic stem cells, Wnt signalling, BMP signalling, Stembyro

## Abstract

Stem-cell-based embryo models have allowed greater insight into peri-implantation mammalian developmental events that are otherwise difficult to manipulate due to the inaccessibility of the early embryo. The rapid development of this field has resulted in the precise roles of frequently used supplements such as N2, B27 and Chiron in driving stem cell lineage commitment not being clearly defined. Here, we investigate the effects of these supplements on embryoid bodies to better understand their roles in stem cell differentiation. We show that Wnt signalling has a general posteriorising effect on stem cell aggregates and directs differentiation towards the mesoderm, as confirmed through the upregulation of posterior and mesodermal markers. N2 and B27 can mitigate these effects and upregulate the expression of anterior markers. To control the Wnt gradient and the subsequent anterior versus posterior fate, we make use of a BMP4 signalling centre and show that aggregates in these conditions express cephalic markers. These findings indicate that there is an intricate balance between various culture supplements and their ability to guide differentiation in stem cell embryo models.

## Teaser

**Summary:** The complex reagents used in ‘stembryo’ protocols have unclear roles in stem cell differentiation *in vitro* requiring further investigation. This study examines their effects on embryoid bodies.

## INTRODUCTION

Successful mammalian development is dependent on precise communication and coordination between embryonic and extraembryonic tissues, leading a small collection of cells to form a highly complex, precisely defined multicellular organism. The study of these intricate morphogenic events is complicated by the fact that the most critical organising events occur during implantation, hidden from view. Implantation-stage development is accompanied by critical cell proliferation, migration, and differentiation events that are crucial to establishing the three germ layers as well as the body axes in a highly co-ordinated process. An understanding of the precise molecular and cellular mechanisms that govern these processes is a fundamental area of study in developmental biology.

The study of the early embryo was for decades reliant on *ex vivo* embryo culture. A large number of studies using various embryo isolation and culture protocols were crucial to gaining a better understanding of the molecular events that govern the shaping of the embryo (Keller, 2013; Bedzhov et al., 2014; Bedzhov and Zernicka-Goetz, 2014; Schrode et al., 2014). However, the requirement for embryos themselves is rate limiting due to the ethical and technical challenges that accompany their use. Recently, however, there has been a significant amount of research into the dynamic self-organising ability of cultured stem cells to recapitulate embryonic development *in vitro* (Turner et al., 2016; Simunovic and Brivanlou, 2017; Shahbazi and Zernicka-Goetz, 2018; Taniguchi et al., 2019; Zylicz, 2020; van den Brink and van Oudenaarden, 2021; Veenvliet et al., 2021; Arias et al., 2022; Amel et al., 2023). These so-called ‘stembryos’ are able to model the events that occur during pre- and peri-implantation development in a remarkably organised and consistent manner. ‘Stembryos’ have thus presented themselves as a powerful tool for investigating the mechanisms underlying early mammalian development, as they allow for unrestricted access and manipulation of developmental events and environmental cues and the tracking of cell fate dynamics without the complications of requiring live embryos.

Rapid progress has thus been made in the ‘stembryo’ field in a very short space of time, with different culture techniques able to resemble the blastocyst (Rivron et al., 2018; Sozen et al., 2019; Yu et al., 2021; Heidari Khoei et al., 2023), initiate pro-amniotic cavity formation and gastrulation (Harrison et al., 2017; Sozen et al., 2018), show signs of anterior–posterior axis elongation (van den Brink et al., 2014; Turner et al., 2017; Beccari et al., 2018), form neural-tube-like structures (Veenvliet et al., 2020; Libby et al., 2021; Berenger-Currias et al., 2022), recapitulate somitogenesis (van den Brink et al., 2020; Veenvliet et al., 2020) and even recapitulate the early stages of cardiogenesis (Rossi et al., 2021; Olmsted and Paluh, 2022). However, with the rapid advancement in this field, the complex nature of the reagents used in these protocols (which are largely shared between the various studies) has left the precise roles of these factors in germ layer differentiation and stem cell self-organisation unclear and requiring further investigation. Reagents such as N2 and B27, both initially designed to aid in the culture of various neuronal cell types and CHIR99021, a Wnt pathway activator commonly known as Chiron, are readily used in a variety of ‘stembryo’ protocols, but their individual roles in driving stem cell lineage commitment as they do in these techniques has not been fully determined. In the present study, we investigate the morphological and gene expression changes induced in embryoid bodies (EBs), the simplest three-dimensional (3D) aggregates of stem cells used to study their differentiation, exposed to commonly used ‘stembryo’ supplements to help dissect their roles in this emerging field.

## RESULTS

### The effect of Chiron on the morphology of mES cell aggregates

**Fig. 1.**
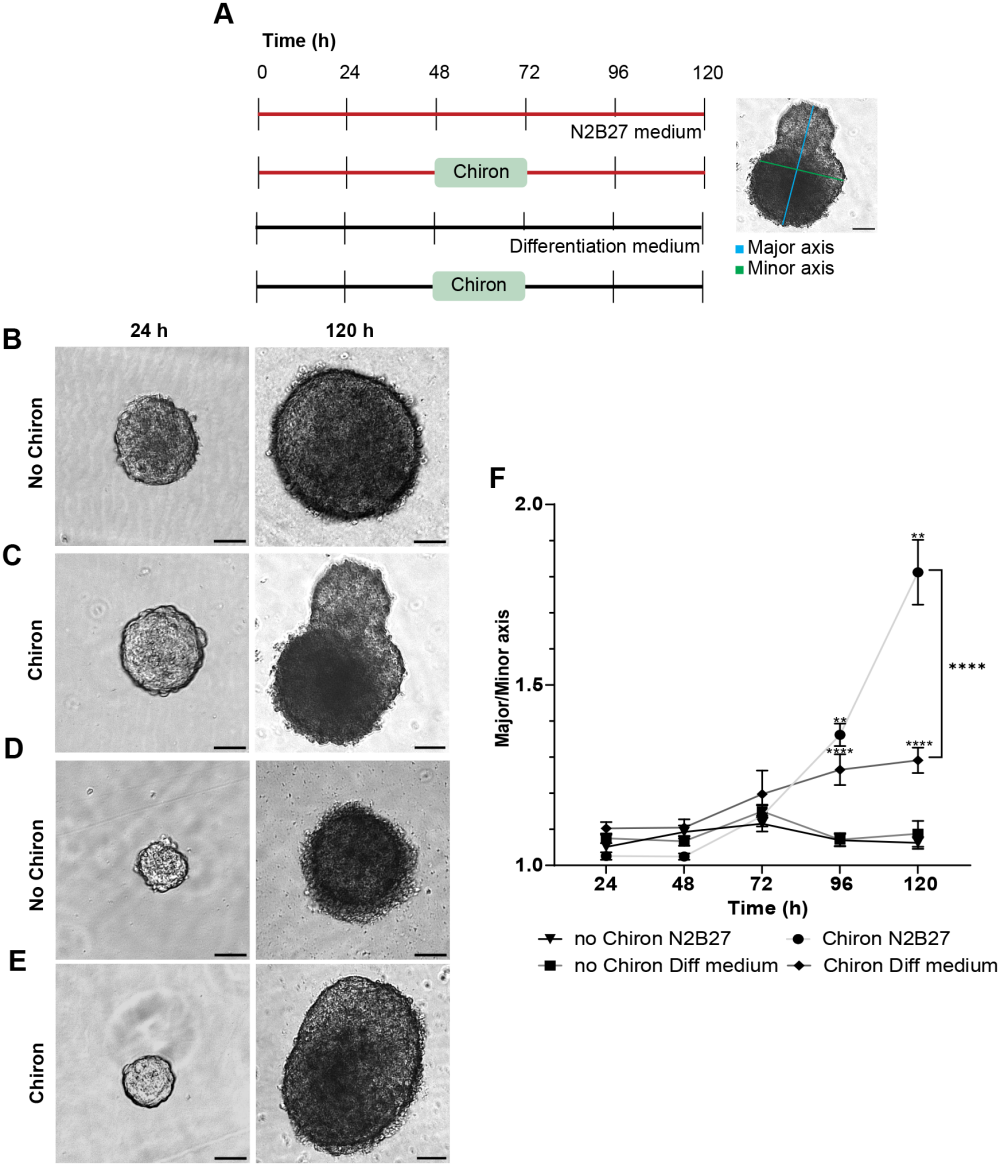
**Comparative analysis of the effect of Chiron on ES cell aggregate morphology.** (A) ES cell aggregates were cultured in several different conditions. In N2 and B27 containing medium with or without a 24 h Chiron pulse and in a standard differentiation medium with or without a 24 h Chiron pulse. Elongation of aggregates was calculated by measuring the longest axis of the aggregate (the major axis; blue line) and the axis perpendicular to this measurement at its midpoint (minor axis; green line). (B) Morphology of aggregates grown in N2B27 medium. Aggregates retained their spherical shape for the duration of 120 h. (C) Morphology of aggregates grown in N2B27 medium that received a Chiron pulse. Aggregates elongated following the Chiron pulse. (D) Morphology of aggregates grown in differentiation medium. Aggregates retained their spherical shape for the duration of 120 h. (E) Morphology of aggregates grown in differentiation medium that received a Chiron pulse. Aggregates elongated slightly following the Chiron pulse. (F) Aspect ratio of aggregates with (grey line, with dots) or without (black line with triangles) a Chiron pulse in N2B27 medium and aggregates with (dark grey line, with diamonds) or without (grey line with squares) a Chiron pulse in differentiation medium. *n*=20 aggregates per condition. Error bars indicate mean±s.e.m. For every time point, an unpaired, nonparametric Mann–Whitney test was performed and **=*P*≤0.0021. Scale bars: 25 µm.

The Wnt agonist Chiron is a commonly used reagent in various ‘stembryo’ culture protocols and is often included in culture via a brief ‘pulse’ together with several other supplements to drive mES differentiation. To examine the role of Chiron in mES cell differentiation we treated aggregates of ES cells cultured in N2B27 with Chiron. Embryoid bodies (EBs) were subjected to a pulse of Chiron at 48 h for 24 h and were compared to EBs without Wnt agonist treatment as well as in the absence of N2B27 (Fig. 1A). In the absence of Chiron in N2B27, the aggregates, regardless of size, remained mostly spherical (Fig. 1B). However, the inclusion of a Chiron pulse while in N2B27 medium resulted in a significant morphological change, as the aggregates elongated into a shape resembling the gastrulating embryo (Fig. 1C). Interestingly, when EBs were subjected to a Chiron pulse in the absence of N2B27 a similar trend was noted with elongation noted post Chiron pulse (Fig. 1D and E). To quantify the shape changes over time, the longest axis of the aggregate (the major axis) was measured, as well as the distance perpendicular to the midpoint of the longest axis (the minor axis) to determine an aspect ratio (Fig. 1A). The aspect ratio of the EBs without Chiron in the presence or absence of N2B27 did not change significantly over the 120 h period, indicating that they retained their spherical shape (Fig. 1F). EBs treated with a Chiron pulse in N2B27 showed a rapid increase in the aspect ratio from 48 h to 96 h, with a significantly more elongated shape compared to those without a Chiron pulse (Fig. 1F, *P*=0.0021). Notably, while a Chiron pulse in the absence of N2B27 was sufficient to drive a quantifiable elongation in aggregates, this response was significantly reduced when compared to cultures in N2B27 (Fig. 1F, *P*<0.001). Additionally, we observed that the shape of the aggregates varied significantly after the Chiron pulse. Aggregates that were noted to be very large as well as very small aggregates were not able to elongate even when exposed to the Wnt agonist, suggesting that the elongation process depends on the size of the aggregates. On the other hand, without a Chiron pulse, the aggregates retained their spherical shape regardless of their initial size.

### A Chiron pulse induces gastrulation and favours mesoderm and endoderm formation

**Fig. 2.**
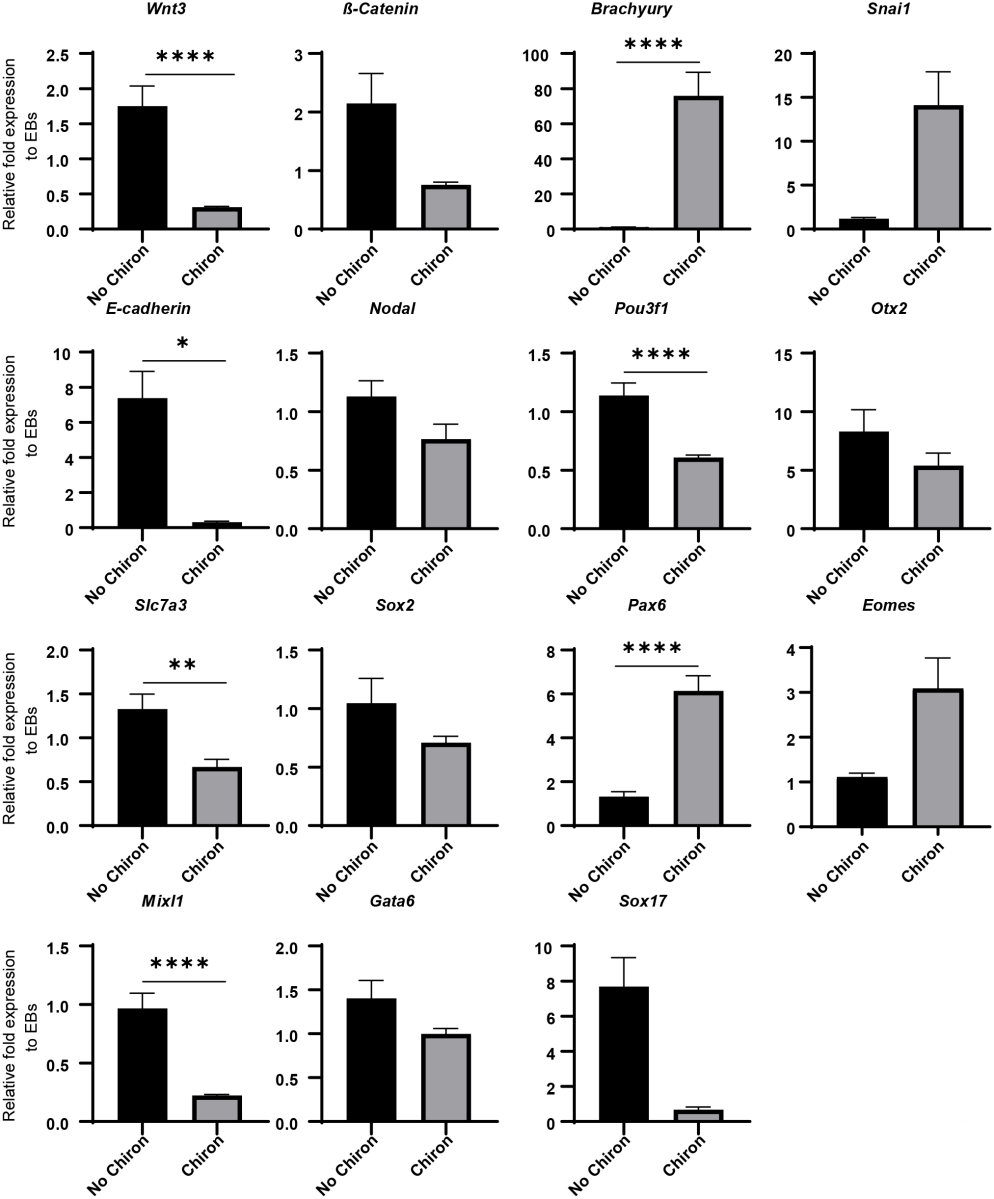
**Effects of a Chiron pulse on gene expression of aggregates grown in differentiation medium.** QPCR was performed on reverse transcribed RNA extracted at 120 h for *n*=2 biological replicates. Primitive streak markers: *Wnt3, Brachyury* and *Nodal.* EMT markers: *E-cadherin, ß-catenin* and *Snai1*. Anterior/neurectoderm markers: *Pou3f1, Otx2, Slc7a3, Sox2* and *Pax 6*. Mesendoderm markers: *Eomes,* and *Mixl1.* Endoderm markers: *Gata6* and *Sox17*. Relative fold expression to EBs without Chiron. Error bars indicate mean±s.e.m. An unpaired, nonparametric Mann–Whitney test was performed and *=*P*≤0.0332, **=*P*≤0.0021, ***=*P*≤0.0002, ****=*P*< 0.0001.

The Wnt pathway plays an essential role in the induction of the primitive streak that breaks the bilateral symmetry of the embryo as epiblast cells undergo an epithelial-to-mesenchymal transition (EMT) and move inward, forming mesodermal and endodermal lineages and marking the posterior of the embryo (Yamaguchi, 2001; Tam and Loebel, 2007; Bardot and Hadjantonakis, 2020). In this study, we sought to determine the degree to which similar events could be induced in EBs via a Wnt agonist pulse. To investigate this, we measured the expression of EMT and primitive streak markers using qPCR in EBs with or without the addition of a Chiron pulse. A pulse of Chiron was sufficient to induce a downregulation of *Wnt3* and *ß-Catenin* expression with upregulation of *Brachyury* and *Snai1 noted* (Fig. 2) indicating the initiation of gastrulation and the EMT process. The upregulation of the mesenchymal marker *Snai1* resulted in a subsequent downregulation of epithelial marker *E-cadherin* and is indicative of an EMT taking place (Fig. 2). Interestingly, no significant difference was noted in *Nodal* expression following a Chiron pulse (Fig. 2). Overall, these results indicate that Chiron is able to drive an EMT in ES cells in culture.

Primitive streak formation is a crucial event in establishing the embryonic germ layers. To assess the degree to which a Wnt agonist can influence lineage commitment, the expression of various germ layer markers was quantified using qPCR with and without a Chiron pulse. Expression levels of anterior/neuroectoderm markers were generally significantly downregulated following a pulse of Chiron, with decreased expression noted in *Pou3f1, Otx2, Slc7a3* and *Sox2* (Fig. 2). This is consistent with the known posteriorising effects of Wnt signalling on the embryo. However, *Pax6,* a key transcription factor in central nervous system development (Osumi et al., 2008), was significantly upregulated following the induction of Wnt signalling (Fig. 2, *P*<0.0001). The mesendoderm marker *Eomes* (Izumi et al., 2007) showed a significant upregulation following a Chiron pulse (Fig. 2). However, another mesendoderm marker *Mixl1* (Izumi et al., 2007) was significantly downregulated following a Wnt pulse (Fig. 2). The endoderm markers *Gata6* and *Sox17* (Morrisey et al., 1998; Kanai-Azuma et al., 2002) were also downregulated, although not significantly (Fig. 2). Overall, these results show that a Chiron pulse is sufficient to drive mesoderm and endoderm differentiation at the expense of ectoderm formation in mES cells.

### N2B27 contrasts posteriorising signals of Chiron

N2 and B27 are critical components of various ‘stembryo’ culture protocols (van den Brink et al., 2014; Harrison et al., 2017; Turner et al., 2017; Beccari et al., 2018), but the precise role of these complex supplements, particularly with the addition of a pulse of Wnt agonist, requires further investigation. To explore the effect of N2B27 on EMT, primitive streak formation, and germ layer induction, we investigated the gene expression of various relevant markers using qPCR in EBs cultured in N2B27 medium with and without a Chiron pulse.

**Fig. 3.**
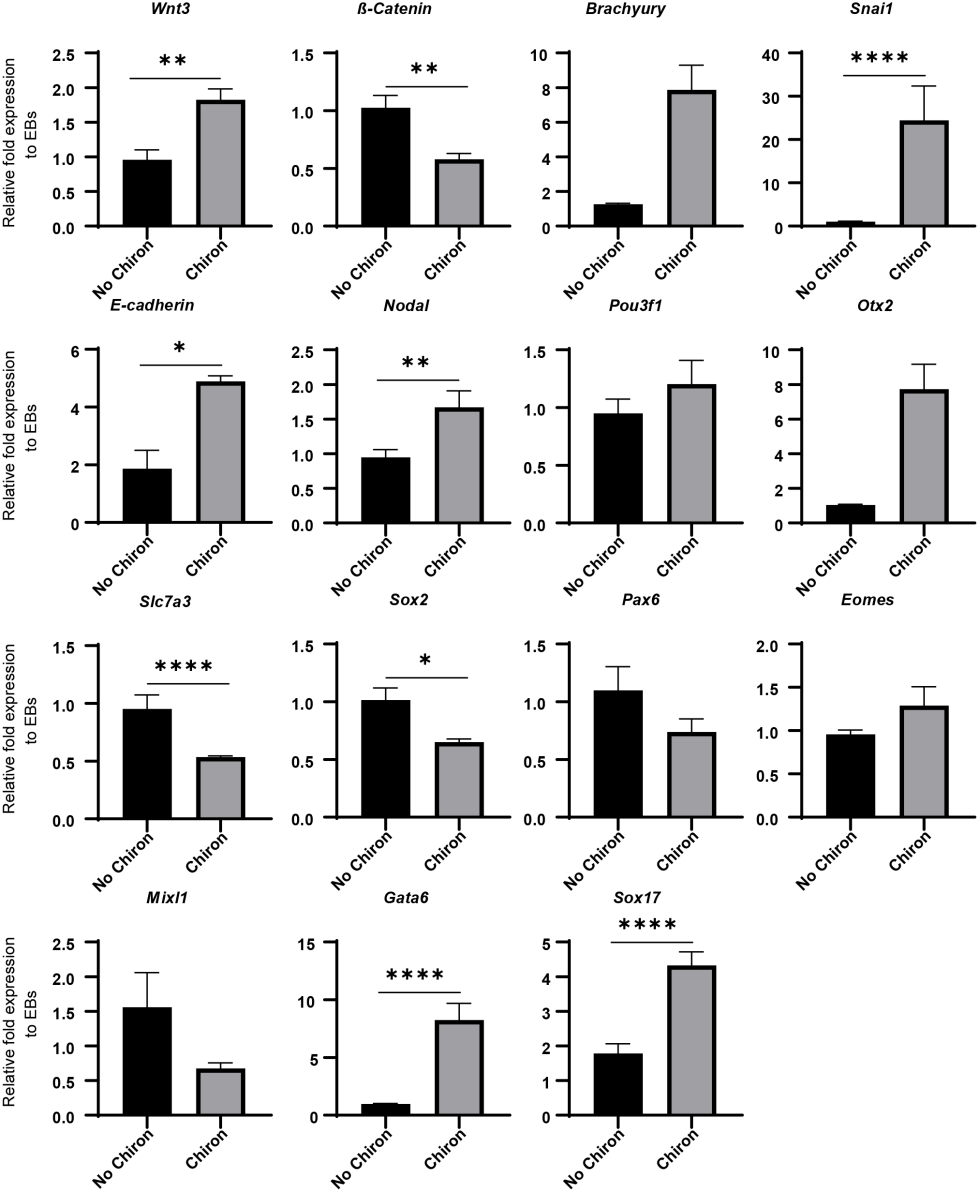
**Effects of a Chiron pulse on gene expression of aggregates cultured in N2B27 medium.** QPCR was performed on reverse transcribed RNA extracted at 120 h for *n*=2 biological replicates. Primitive streak markers: *Wnt3, Brachyury* and *Nodal.* EMT markers: *E-cadherin*, *ß-catenin* and *Snai1.* Anterior/neurectoderm markers: *Pou3f1, Otx2, Slc7a3, Sox2* and *Pax6.* Mesendoderm markers: *Eomes* and *Mixl1*. Endoderm markers: *Gata6* and *Sox17*. Relative fold expression normalised to EBs without Chiron. Error bars indicate mean±s.e.m. An unpaired, nonparametric Mann–Whitney test was performed and *=*P*≤0.0332, **=*P*≤0.0021, ***=*P*≤0.0002, ****=*P*< 0.0001.

The addition of a Chiron pulse had a different effect on *Wnt3* expression in the presence and absence of N2 and B27. In the presence of N2B27 medium, *Wnt3* expression was significantly upregulated by the Chiron pulse (Fig. 3), while it was downregulated without N2B27 medium (Fig. 2). While a downregulation of *ß-catenin* and an upregulation of *Brachyury* was observed following a Chiron pulse in N2B27 medium (Fig. 3) similar to what was seen before (Fig. 2), the fold expression was significantly reduced in N2B27 containing medium. Both *Nodal*, a primitive streak and mesoderm marker (Conlon et al., 1994), and *E-cadherin*, an epithelial marker, were significantly upregulated following a Chiron pulse in N2B27 medium (Fig. 3), again contrasting the expression changes seen without N2 and B27 (Fig. 2). There was a significant upregulation of the EMT marker, *Snai1,* following a pulse of the Wnt agonist in N2B27 medium (Fig. 3) coinciding with the effect of Chiron on *Snai1* seen before (Fig. 2).

There was a significant upregulation of the early anterior marker *Otx2* (Perea-Gomez et al., 2001) (Fig. 3) and a downregulation of both *Slc7a3* and *Sox2*, markers of the anterior epiblast (Peng et al., 2016; Osteil et al., 2019; Probst et al., 2021) and neural stem cells (Zhang, 2014), respectively, following a Chiron pulse in N2B27 medium. There were no significant effects on *Pou3f1* and *Pax6* expression following a Chiron pulse in N2B27 medium (Fig. 3). Both mesendoderm markers, *Eomes* and *Mixl1*, did not have any significant differences in expression in N2B27 medium following a Chiron pulse (Fig. 3) although it was noted that the trend of both these markers was similar to what was seen in the absence of N2 and B27 following Wnt signalling (Fig. 2). Interestingly, the expression of both endoderm markers investigated, *Gata6* and *Sox17,* was significantly upregulated following Wnt induction in N2B27 medium (Fig. 3). Together, these results suggest that N2 and B27 may be working in opposition to the posteriorising effects of Chiron, eliciting an anteriorising signal in mES aggregates. Combining N2, B27, and Chiron in mES differentiation protocols therefore allows for the simultaneous generation of lineages from different germ layers as is required when forming a ‘stembryo’.

### BMP4 has contrasting effects to Wnt signalling on the elongation of ES cell aggregates in culture

**Fig. 4.**
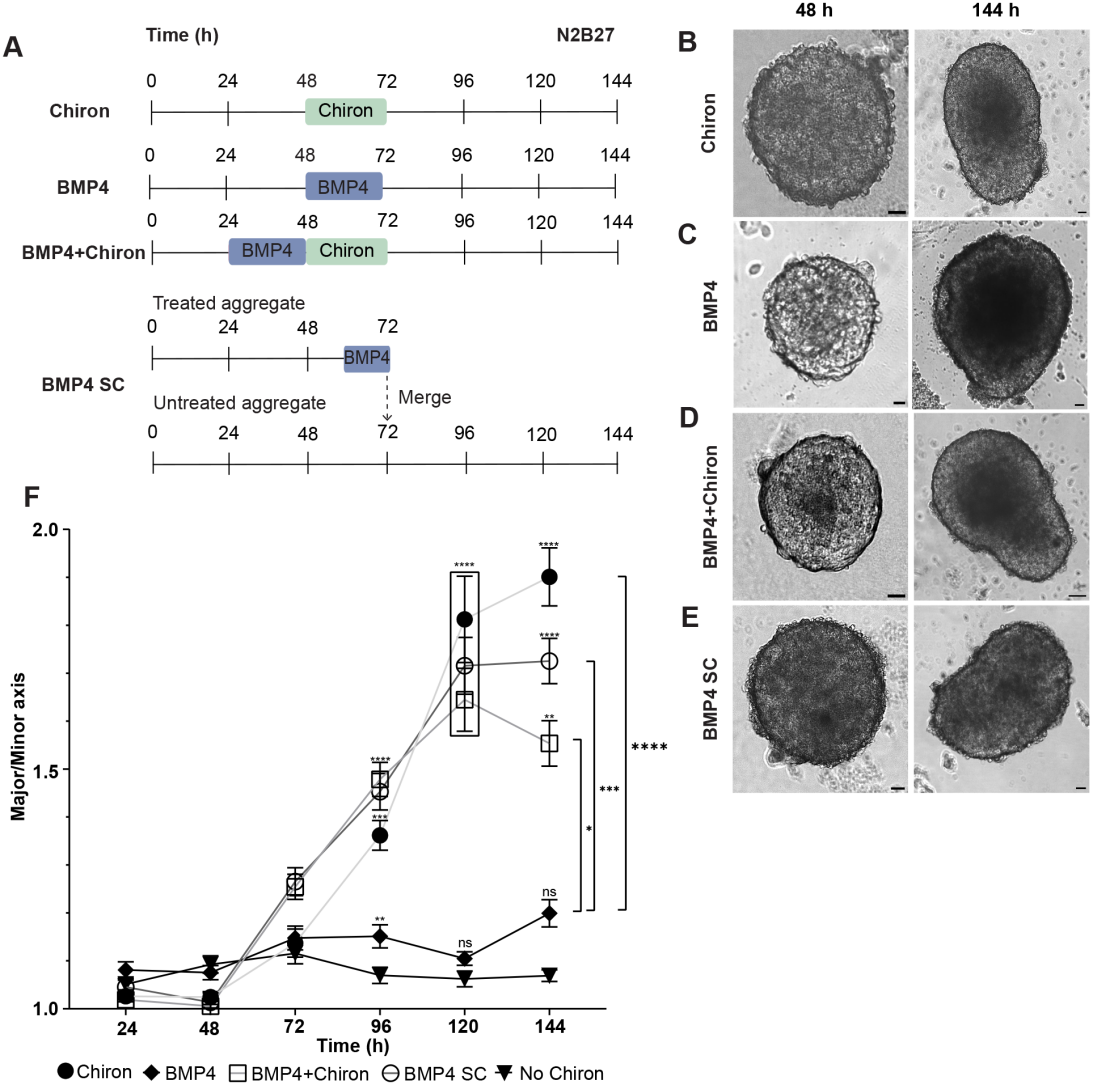
**Comparative analysis of the effect of BMP4 on the ES cell aggregate morphology.** (A) ES cell aggregates were cultured in several different conditions. Chiron aggregates were cultured in N2B27 medium and received a 24 h Chiron pulse on the second day of culture. BMP4 aggregates were cultured in N2B27 medium and received a 24 h BMP4 pulse on the second day of culture. BMP4+Chiron aggregates received a 24 h BMP4 pulse followed by a 24 h Chiron pulse. BMP SC aggregates were made by merging a smaller aggregate, which had been treated with BMP4 for 8 h, with a larger, untreated aggregate on the third day of culture. (B) Morphology of Chiron aggregates at 48 h and 144 h. Aggregates elongated following the Chiron pulse. (C) Morphology of BMP4 aggregates at 48 h and 144 h. Aggregates remained approximately spherical following the BMP4 pulse (D) Morphology of BMP4+Chiron aggregates at 48 h and 144 h. Aggregates were able to elongate effectively but not as much as Chiron aggregates. (E) Morphology of BMP4 SC aggregates at 48 and 144 h. Aggregates were able to elongate but retained an oval shape. (F) Aspect ratio of Chiron (light-grey line, with black circles), BMP (black line, with black diamonds), BMP4+Chiron (grey line with open squares), BMP SC (grey line with open circles), No Chiron in N2B27 (black line with black triangle) aggregates for *n*=15 aggregates per condition. Error bars indicate mean±s.e.m. A one-way ANOVA with Dunnett's multiple comparisons post-test was carried out and *=*P*≤0.0332, **=*P*≤0.0021, ***=*P*≤0.0002, ****=*P*< 0.0001. Scale bars: 25 µm.

BMP4 is crucial for initiating anterior–posterior patterning and gastrulation in the mouse embryo by enhancing Wnt expression (Arnold and Robertson, 2009; Bardot and Hadjantonakis, 2020). Recently, it has been shown that exposure to a BMP4 signalling centre is sufficient to drive embryonic morphogenesis in culture (Xu et al., 2021). As such, we next wished to examine the role of BMP4 signalling on mES cell differentiation. We treated ES cell aggregates with a pulse of BMP4, pulses of both BMP4 and Chiron (BMP4+Chiron), or combined ES aggregates with a BMP4 signalling centre (BMP4 SC) (Fig. 4A). The BMP SC was created by adding BMP4 to a small aggregate of mESCs for 8 h, after which time these BMP4 exposed aggregates were merged with untreated larger aggregates to create a combined aggregate with an effective BMP4 exposed pole. We compared the aggregates with EBs treated with just a Chiron pulse as above, with all culture taking place in N2B27 containing medium.

At 48 h, all aggregates were roughly spherical in shape and had doubled from initial seeding size (Fig. 4B-E). EBs exposed to Chiron began to elongate by 72 h and continued to do so throughout the culture period (Fig. 4B and F). In contrast, EBs exposed to a pulse of BMP4 retained their approximately spherical shape throughout the culture period (Fig. 4C and F). EBs treated with pulses of both BMP4 and Chiron broke symmetry and elongated (Fig. 4D). However, there was a significant reduction in aspect ratio compared to those treated with Chiron alone by 144 h (Fig. 4F). Similarly, EBs treated with a BMP4 signalling centre were also found to be more oval in shape than those treated with a Chiron pulse (Fig. 4E) with a significantly reduced aspect ratio compared to a Chiron-only pulse by 144 h (Fig. 4F). Interestingly, while by 144 h the Chiron aggregates had, as mentioned, elongated more than the other conditions, the aspect ratio of BMP4 aggregates (both those receiving a pulse or a signalling centre) was significantly higher at 72 h and 96 h when compared to the Chiron aggregates (Fig. 4E). These results suggest that BMP4 and Wnt signalling have contrasting roles to play in the rate and degree to which ES cell aggregates can elongate.

### Localised BMP4 signalling allows the expression of anterior neural markers in cell aggregates

**Fig. 5.**
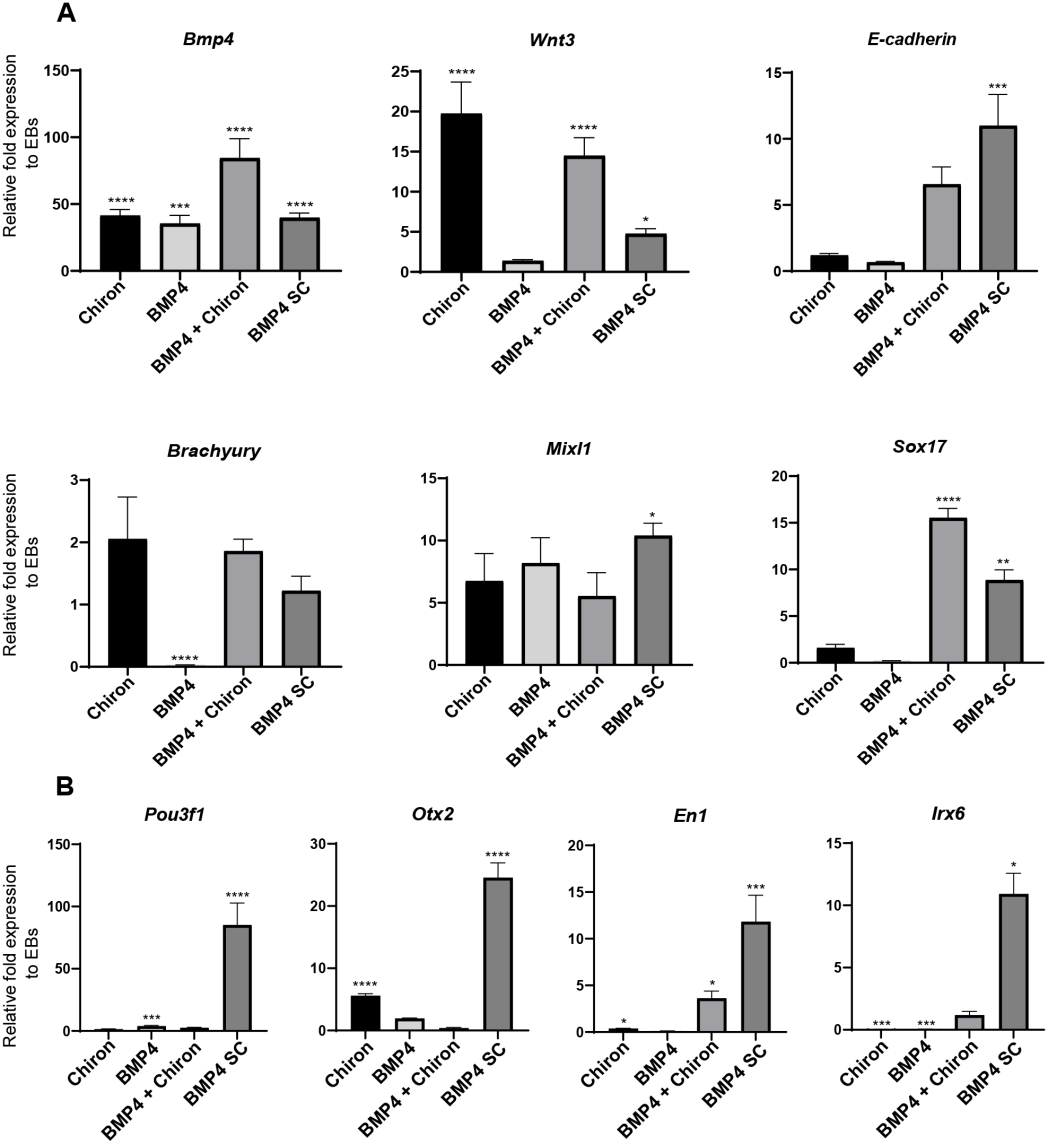
**Effects of BMP4 on gene expression of aggregates.** (A) QPCR was performed on reverse transcribed RNA extracted at 144 h for *n*=2 biological replicates. Posterior and primitive streak markers: *BMP4* and *Wnt3*. EMT marker: *E-cadherin*, mesoderm markers: *Brachyury* and *Mixl1* and endoderm marker: *Sox17*. (B) Anterior neuroectodermal markers were highly expressed in BMP4 SC aggregates. Relative fold expression normalised to EBs without Chiron. Error bars indicate mean±s.e.m. A one-way ANOVA with Dunnett's multiple comparisons post-test was carried out and *=*P*≤0.0332, **=*P*≤0.0021, ***=*P*≤0.0002, ****=*P*< 0.0001.

As the inclusion of BMP4 had a clear effect on ES aggregate morphology, we next investigated the effects of our culture conditions on the expression of various germ layer markers, using qPCR. Exogenous BMP4 significantly increased *Bmp4* mRNA expression under BMP4+Chiron conditions as expected (Fig. 5A). Interestingly, BMP4 pulse and SC aggregates had similar levels of *Bmp4* mRNA as the Chiron treated aggregates, despite the latter not receiving any exogenous BMP4 (Fig. 5A). Both conditions exposed to a Wnt agonist showed a highly significant increase in *Wnt3* mRNA expression as expected, in contrast to BMP4 pulse and SC aggregates which had significantly lower levels of *Wnt3* compared to Wnt agonist receiving aggregates (Fig. 5A). Interestingly, while a BMP4 or Chiron pulse individually did not upregulate levels of *E-cadherin*, BMP4+Chiron and the BMP4 SC conditions both had increased *E-cadherin* with levels in the BMP4 SC significantly upregulated (Fig. 5A). *Brachyury* was expressed in all conditions except in the BMP4 pulse, while *Mixl1* mRNA was found in all conditions without high levels of variation (Fig. 5A). While Sox17 was not highly expressed in the individual BMP4 or Chiron pulse conditions, BMP4+Chiron and the BMP4 SC conditions both had significantly increased levels of *Sox17* (Fig. 5A).

Most strikingly, all the anterior neural markers investigated, *Pou3f1, Otx2, En1, and Irx6,* were all highly expressed in BMP SC cultures with this same effect not being observed under conditions with a BMP4 pulse (Fig. 5B).

## DISCUSSION

The ability of stem cells to self-organise in culture and form complex, embryo-like structures presents a non-invasive, scalable, and ethically viable way to investigate gastrulation and germ layer formation in mammals. Many ‘stembryo’ culture systems make use of N2 and B27 (van den Brink et al., 2014; Baillie-Johnson et al., 2015; Turner et al., 2017; Beccari et al., 2018), supplements originally designed to specifically support neuronal cell culture (Bottenstein and Sato, 1979; Brewer et al., 1993). B27, which contains retinyl acetate, is particularly important for the formation of neuronal tissue in the posterior hindbrain and the anterior region of the spinal cord (Brewer et al., 1993; Ross et al., 2000), while N2 contains insulin, selenium and transferrin and has been shown to support neurogenesis (Bottenstein and Sato, 1979).When mES cells are cultured in medium containing both N2 and B27 they differentiate towards a neural fate (Ying et al., 2003; Abranches et al., 2013). ‘Stembryo’ culture protocols also make liberal use of a potent Wnt agonist CHI99021, commonly known as Chiron, in a pulsatile manner (van den Brink et al., 2014; Baillie-Johnson et al., 2015; Turner et al., 2017; Beccari et al., 2018). Wnt signalling is known to be involved in initiating gastrulation through up-regulation of the primitive streak marker Brachyury in the embryo (Dunty et al., 2008; Wang et al., 2012; Arkell et al., 2013) and has a similar effect in ‘stembryo’ culture (van den Brink et al., 2014; Baillie-Johnson et al., 2015; Turner et al., 2017; Beccari et al., 2018). BMP4 is also critical for the initiation of the primitive streak. BMP4 from the extraembryonic ectoderm upregulates the expression of Wnt3 and Nodal in the posterior region of the epiblast, which initiates this symmetry breaking process and creates a feed-forward signalling complex (Perea-Gomez et al., 2004; Rivera-Perez and Magnuson, 2005; Arnold and Robertson, 2009). The expression of these signalling molecules is restricted to the posterior region by antagonists expressed in the anterior epiblast (Thomas and Beddington, 1996; Kumar et al., 2015; Antonica et al., 2019). Under the influence of anterior transcription factors, this region will form the neural plate, giving rise to the brain. Treating stem-cell aggregates with BMP4 has been shown to induce the expression of Wnt and Nodal (Xu et al., 2021). When these BMP4 aggregates are merged with a second, larger, untreated aggregate, they are able to act as a signalling centre to initiate gastrulation in the now merged ‘stembyro’ (Xu et al., 2021). The combination of these various signalling pathways, induced in stem cells using a variety of supplements and ligands, has resulted in the rapid development of various ‘stembryo’ culture systems. This novel field continues to deliver critical insights into the earliest organisational events that occur during mammalian development in a short period of time. However, the rapid development of this field has resulted in the precise effects of some of these various culture supplements on stem cell differentiation remaining undefined. Here, we treated EBs with various ‘stembryo’ culture reagents to begin investigating their precise effects on stem-cell aggregate morphology and gene expression.

Our results show that Chiron makes stem-cell aggregate elongation a more robust process, an observation supported by other studies (van den Brink et al., 2014; Turner et al., 2017). We also observed that Chiron is significantly more effective in inducing elongation when aggregates are cultured in the presence of N2 and B27. Interestingly, BMP4 hindered the elongation of stem-cell aggregates, even in the presence of Chiron. This is consistent with previous studies that found that BMP4 can inhibit axial elongation in the mouse embryo (Turner et al., 2017; Girgin et al., 2021).

We found that the addition of Chiron resulted in a decrease in *Wnt3* and *ß-catenin* mRNA in differentiation medium. Chiron is a Gsk3 inhibitor that prevents degradation of *ß-catenin*, which is able to accumulate and act on downstream targets of the Wnt3 pathway thereby serving as a Wnt pathway agonist. We hypothesise that the activation of the Wnt pathway and accumulation of its downstream targets initiates a negative feedback loop resulting in downregulation of *Wnt3* and *ß-catenin* in differentiation medium. As BMP4 from the extraembryonic ectoderm enhances Wnt expression in the epiblast (Liu et al., 1999; Tam and Loebel, 2007; Arnold and Robertson, 2009) we expected that addition of BMP4 together with Chiron would increase the expression of *Wnt3* mRNA in mES cells. However, although EBs treated with both BMP4 and Chiron did have significantly higher levels of *Wnt3* mRNA compared to untreated EBs, *Wnt3* expression was reduced compared to aggregates treated with just Chiron, indicating that BMP4 had hindered *Wnt3* expression in differentiation cultures*.* Chiron aggregates were also found to have high levels of *Bmp4* compared to untreated aggregates even in the absence of exogenous BMP4 treatment. It may be that Wnt3 activation, through a Chiron pulse, resulted in an increase in *Bmp4* expression, mimicking the BMP4/Nodal/Wnt signalling complex that occurs in the posterior proximal region of the embryo during primitive streak formation and gastrulation (Liu et al., 1999; Tam and Loebel, 2007; Arnold and Robertson, 2009). Similarly, it can be argued that the signalling centre treated with BMP4 under BMP SC conditions, which has been shown to have an induced expression of Nodal and Wnt3 (Xu et al., 2021), interacts with the untreated aggregate and induces the expression of *Bmp4*.

In our study, the addition of Chiron was able to downregulate *E-cadherin* expression and upregulate *Snai1* expression as occurs in the embryo during EMT (Kim et al., 2017), thereby suggesting that Chiron facilitates this process in mES cell aggregates. Chiron acted as a posteriorising signal directing differentiation towards the mesendoderm lineage, as observed with decreased expression of anterior markers and increased expression of the primitive streak marker and mesendoderm markers *Brachyury* and *Eomes*. We also observed a decrease in the endoderm markers *Sox17* and *Gata6* after the EBs were treated with a Chiron pulse, which may suggest that Chiron directs mesendoderm progenitors towards the mesoderm rather than the endoderm lineage (Ying et al., 2008), although we should expect to see endoderm progenitors in our structures (van den Brink et al., 2014; Turner et al., 2017). We found that aggregates exposed to either a BMP4 pulse or signalling had a significant increase in the expression of the definitive endoderm marker Sox17, and this is supported by previous work that has shown that BMP4 functions to specify the definitive endoderm lineage (Teo et al., 2011; 2014). The definitive endoderm forms an epithelial layer that covers the epiblast and is associated with high levels of E-cadherin (Nowotschin et al., 2019). Studies have shown that stem cell aggregates form an endoderm-like layer on their surface and these cells co-express Sox17 and E-cadherin, resembling the definitive endoderm forming on the outside of the mouse embryo (van den Brink et al., 2014; Hashmi et al., 2022). It follows that BMP treatment of EBs is driving differentiation towards the endoderm lineage.

Interestingly, *Pax6*, a gene involved in the formation of the central nervous system (Osumi et al., 2008), was upregulated in our Chiron-treated aggregates. Various studies have shown that Wnt signalling upregulates *Pax6* expression in the mouse brain and its outgrowths, including the cerebellum (Yeung et al., 2016), the optic cup (Canto-Soler and Adler, 2006), and the olfactory epithelium (Collinson et al., 2003). Although our aggregates have not reached these advanced stages of development, increased expression of *Pax6* may be an indicator of precursor cells that would eventually develop into these features.

Wnt signalling plays a broad role in the developing mouse embryo and regulates different downstream effectors depending on the tissue and stage of development. In our study, we found that Chiron acts downstream of the Wnt/ß-catenin pathway and serves to make elongation a more reproducible event in mES cell aggregates. As a result of Chiron signalling, Brachyury expression is upregulated, and the primitive streak is initiated. Differentiation is primarily directed toward the mesoderm lineage as a result of activated Wnt signalling, however, due to the complex role of the Wnt signalling pathway in differentiation, there may be an increase in expression of non-mesodermal markers, such as Pax6. We also hypothesise that Chiron, as a downstream effector of the Wnt pathway, may regulate the expression of Wnt proteins through a negative feedback loop. We found that BMP4, which, in the embryo, acts to enhance the effects of Wnt signalling, appears to hinder the effect of Chiron by preventing the elongation of aggregates that have been subjected to a Chiron pulse. In contrast to the mesoderm-inducing effects of Chiron, BMP4 favours endoderm differentiation. Mesoderm and endoderm cells share a common precursor: mesendoderm cells. In the embryo, the path of differentiation is dictated by the concentration of various signalling molecules, including BMP4 and Nodal (Vincent et al., 2003; Bardot and Hadjantonakis, 2020). To faithfully recreate these events *in vitro*, optimisation of the dosage and timing of exogenous factors such as Chiron and BMP4 is needed in order to recapitulate the body plan in mES cells.

Cell aggregates exposed to high Wnt signalling, in the form of a Chiron pulse, lack brain and head structures, and resemble the post-occipital region of embryos (van den Brink et al., 2014; Turner et al., 2017; Beccari et al., 2018). We found that the anterior markers *Pou3f1*, *Otx2* and *Slc7a3* were downregulated with the addition of a Chiron pulse in our EBs, thereby suggesting that Chiron hindered anterior neural fate commitment. On the other hand, it has previously been shown that when mES cell monolayers are cultured in N2B27 medium they differentiate into glial subtypes such as astrocytes and oligodendrocytes (Ying et al., 2003; Abranches et al., 2009). Therefore, we cultured EBs in N2B27 medium with and without a Chiron pulse to investigate if this would balance anterior versus posterior differentiation in our stem cell aggregates and recreate a more complete body plan with anterior and posterior structures. We noted an increase in *Wnt3* expression after a Chiron pulse in N2B27 medium, in contrast to the effect of Chiron on *Wnt3* without the presence of N2 or B27. We hypothesise that this reversal of effect is due to the complex nature of the cell culture medium, with various components, such as retinyl acetate in B27, that can modulate the response of cells to Chiron and result in an increase in *Wnt3* expression. We hypothesised that since Chiron acts downstream of the Wnt/ß-catenin pathway, it is able to upregulate the expression of downstream targets of this pathway, which, in turn, work in a negative feedback loop to downregulate the expression of *Wnt3*. Studies have shown that retinoic acid (a retinyl acetate metabolite) inhibits downstream Wnt/ß-catenin events (Hu et al., 2013; Osei-Sarfo and Gudas, 2014) but increases the expression of canonical Wnt ligands: Wnt2, Wnt3a and Wnt8a (Osei-Sarfo and Gudas, 2014). Therefore, it may be possible that the retinyl acetate in our medium reduces the expression of downstream targets of the Wnt pathway, which would affect the regulatory loop and result in an upregulation of the Wnt3 gene. *Brachyury* remained upregulated in the presence of N2B27 and Chiron, although not as significantly as expression in the absence of N2B27, suggesting that N2B27 may balance the expression of mesoderm or posterior markers with anteriorising signals (Thomson et al., 2011; Turner et al., 2014b) possibly from the effects of retinyl that is essential for neocortex formation (Haushalter et al., 2017) . We observed an increase in the expression of the anterior epiblast markers *Pou3f1* and *Otx2* in the presence of N2B27 and Chiron, contradicting the assumption that Chiron increases the expression of posterior markers and decreases the expression of anterior markers (Turner et al., 2014a). It may be that N2 and B27 reverse the posteriorising effects of Chiron by enhancing the expression of anterior markers. The results suggest that the addition of Chiron to stem cell aggregates promotes posterior neural fate commitment and inhibits the development of brain and head structures. However, the use of N2B27 medium appears to balance the expression of posterior markers with anteriorizing signals, resulting in the expression of anterior markers. Therefore, tightly regulating the timing and specific anteriorising and posteriorising signals in ‘stembyro’ culture systems will allow the recreation of a more complex *in vitro* embryo model.

We found that introducing BMP4 as a spatially restricted signalling centre in aggregates resulted in significant up-regulation of neural markers, including *Pou3f1, En1, Irx6* and *Otx2*. There was a notable and distinct increase in all of these markers in the BMP4 SC aggregates compared to those that just received a BMP4 pulse. This is significant evidence that it is not only the concentration at which supplements are added to cell culture that is critical, but the way they are introduced that can direct stem cell differentiation in ‘stembryo’ models. The increase in the expression of anterior neural markers under BMP4 SC conditions confirms the ability of this model to drive anterior embryonic differentiation. In this model, the signalling centre is exposed to BMP4, which induces the expression of Nodal and Wnt3. Once this signalling centre merges with the untreated aggregate, it serves as the posterior pole of the aggregate with high concentrations of Nodal and Wnt3, as occurs in the embryo. A morphogen gradient is created, and the region farthest away from the signalling centre will receive the least Nodal and Wnt3 activity and will serve as the anterior pole. In the embryo, the anterior visceral endoderm is responsible for secreting the Nodal and Wnt3 antagonists, Dkk1, Cer1, and Lefty, which restrict the activity of Nodal and Wnt to the posterior region (Arnold and Robertson, 2009; Arkell and Tam, 2012). This model does not contain the anterior visceral endoderm and is based solely on the engineered signalling gradient and serves to demonstrate the importance of a morphogen gradient in creating the body's axes. However, we cannot ignore the roles of Dkk1, Cer1, and Lefty1 in development, and future studies should attempt to create a morphogen gradient of these anterior markers in stem-cell-based models and combine anterior and posterior signalling molecules in one system.

A notable limitation of our study is the prevalence of FBS in our culture medium. Besides the fact that FBS contains undefined growth factors and may be subject to batch-to-batch variability, it has also been shown that EBs cultured in serum-containing medium have been shown to have a localised expression of Wnt and Nodal, which in turn results in increased Brachyury (Ten Berge et al., 2008). FBS also contains BMP4, which may contribute to the induction of Brachyury expression and the initiation of primitive streak formation and gastrulation in the absence of Chiron. Future ‘stembryo’ studies should focus on excluding undefined components and replacing serum entirely to improve experimental reproducibility. While beyond the scope of this study, future work will test the effects of N2 and B27 individually on stem cell morphology and gene expression especially on how this will affect anterior/neuroectoderm markers. N2 and B27 are consistently used together in ‘stembryo’ cultures, and it would be interesting to note their effects when segregated.

Our work described here provides an examination of how common ‘stembryo’ culture reagents, Chiron, N2, B27, and BMP4 signalling impact the morphology and gene expression of stem cells in the simplest 3D differentiation model. Specifically, we found that Chiron treatment of mES cells makes EMT and gastrulation more reproducible, that the Wnt pathway plays a critical role in the posterior region of the embryo during primitive streak formation, and that N2B27 medium can counterbalance the posteriorising effects of the Wnt pathway and promote lineage commitment to the neuroectoderm. Critically, we find that a group of localised cells exposed to BMP4 can effectively drive neural differentiation in stem cell aggregates. Finally, the use of a signalling centre to establish a morphogen gradient can lead to the more prominent establishment of anterior and posterior identity, resulting in a more accurate representation of the developing body and could be a key technique in the ‘stembryo’ field moving forward.

## MATERIALS AND METHODS

### mES cell culture

A feeder layer of inactivated murine embryonic fibroblast cells was cultured in six-well plates on gelatin (0.1%, Sigma-Aldrich G7041-100G) at a seeding density of 1.6×10^4^ cells/cm^2^ in base medium consisting of DMEM (1x) +GlutaMAX™ (Gibco 10566016), 15% FBS (Gibco 10493106), 0.05 mM β-mercaptoethanol (Gibco 21985023), 1% PenStrep (100 U penicillin/ 0.1 mg/ml streptomycin, Gibco 15140122). Two h prior to seeding mES cells (from 129/Ola mice courtesy of Professor Frank Brombacher (Institute of Infectious Diseases and Molecular Medicine, Faculty of Health Sciences, University of Cape Town, Cape Town, South Africa), the medium was changed to the same base medium with the addition of CHIR99021 (Chiron, 3 µM, Sigma-Aldrich SML1046-5MG), PD0325901 (1 μM, Sigma-Aldrich PZ0162-5MG) and Leukaemia inhibitory factor (10 ng/ml, LIF, Thermo Fisher Scientific A35934). 129/Ola mES cells were seeded on the feeder layer at a density of 7×10^3^ cells/cm^2^. Medium was changed daily, and cells passaged every other day.

### mES differentiation

mES colonies were treated with Dispase II (5 mg/ml, Sigma-Aldrich D4693-1G) at 37°C for 20 min, followed by inactivation of Dispase and transfer of the cell suspension to a 15 ml tube. The cells were left to settle at the bottom for 10 min, after which time the supernatant was removed and the cells were resuspended in base medium, referred to as differentiation medium, or N2B27, which is made up of differentiation medium supplemented with 1% N2 supplement (Gibco 17502048), 1% B27 plus supplement. (Gibco A3582801). The cells were triturated gently to break the clusters into smaller aggregates. The contents of the tube were then transferred to a 6 cm bacteriological plate and an additional 5 ml of differentiation medium or N2B27 was added to the plate. Alternatively, to form individual aggregates, dissociated mES cells were resuspended at a concentration of 6.25×10^3^ cells/ml in N2B27 and 40 µl droplets of were pipetted into each well of a non-tissue-culture treated U-bottomed 96-well plate (Greiner Bio-One 650185). For experiments requiring a Chiron pulse, CHIR99021 was added at a final concentration of 3 µM as added 48 h to 72 h post aggregate formation. For experiments with a BMP and Chiron pulse, 24 h post individual aggregate formation, BMP4 (Gibco PHC9534) was added to each well of the U-bottomed 96-well plate at a final concentration of 1 ng/ml. After 24 h, the medium was replaced with N2B27 supplemented with Chiron at a final concentration of 3 µM. To create a BMP signalling centre, 40 μl droplets of a suspension of 3.75×10^3^ mESC cells/ml in N2B27 medium were pipetted into each well of a U-bottomed 96-well plate and left to settle for 40 h, after which, BMP4 at a final concentration of 1 ng/ml was introduced into each well for a total of 8 h. Simultaneously, a second aggregate of mES cells was created by placing 40 μl droplets of a 2.5×10^4^ mES cells/ml suspension in N2B27 medium pipetted into each well of a U-bottomed 96-well plate and left to settle for 48 h. Then, both these aggregates were merged 48 h after their initial formation.

### RNA extraction and cDNA synthesis

RNA was extracted at 120 h or 144 h post aggregation using the High Pure RNA Isolation Kit (Roche 11828665001) according to the manufacturer's instructions. Due to the limited amount of material isolated from each experiment, two experiments were pooled to create each of the biological replicates. The ImProm-II™ Reverse Transcription System (Promega) was used for cDNA synthesis. Briefly, a minimum of 1 µg of RNA was combined with 1 µl of Oligo (dT)15 primer (Promega, C110B) and nuclease-free water to make up a total of 5 µl and incubated at 70°C and 4°C for 5 min each. PCR master mix was added to each sample: 6.1 µl nuclease-free water, 4 µl 5× reaction buffer (Promega, M289A), 2.4 µl MgCl2 (Promega, A351H), 1 µl dNTP mix (Promega, C114B), 0.5 µl Recombinant RNasin^®^ Ribonuclease Inhibitor (Promega, N251A), 1 µl Reverse Transcriptase (Promega, M314A). Thermal cycling was as follows: 25°C for 5 min, 42°C for 60 min and 70°C for 15 min.

### qPCR

Quantitative PCR was performed on the StepOnePlus™ Real-Time PCR System using SYBR green PCR Master-Mix (ThermoFisher Scientific, 4368708). Primer sequences can be found in supplementary material Table S1. Briefly, 2 µl of cDNA (1:1 dilution with nuclease-free water) was mixed with 8 µl master which consisted of: 5 µl SYBR green Master-Mix, 0.4 µl of 10 µM forward and reverse primer mixture (5 µl of 100 µM stock with 40 µl nuclease-free water), and 2.6 µl nuclease-free water. Each reaction had three technical replicates; see supplementary material Table S2 for run parameters. *Gapdh* was used as a housekeeping gene to calculate relative expression via the 2-ΔΔCt method. Expression was normalised to EBs cultured in either differentiation medium or N2B27 medium that did not receive a Chiron pulse. MS Excel was used for data analysis, and GraphPad Prism8 was used for statistical analysis and graph generation.

### Image analysis

Images were taken using EVOS™ M5000 Imaging System microscope (ThermoFisher Scientific) at 10X magnification. All images were processed using ImageJ. For morphology measurements, the outlines of the entire aggregate were traced manually. The minor and major axial lengths were determined using the line tool. The elongation index was calculated by dividing the major axial length by the minor axial length.

### Statistical analysis

All statistical analyses were performed using the built-in functions of GraphPad Prism 8.4.2 (679). Statistical analysis of the RT-qPCR data was performed on two pooled biological replicates due to limited biological material. When appropriate, an unpaired, nonparametric Mann–Whitney test was performed. Alternatively, a one-way ANOVA with Dunnett's Multiple Comparison's post-test was carried out. In all cases, the error bars represent the mean±standard error of the mean (mean±s.e.m.). *P* values are represented as follows: ns=*P*≤0.1234, *=*P*≤0.0332, **=*P*≤0.0021, ***=*P*≤0.0002, ****=*P*< 0.0001.

## Supplementary Material

10.1242/biolopen.059981_sup1 Supplementary information

## References

[BIO059981C1] Abranches, E., Silva, M., Pradier, L., Schulz, H., Hummel, O., Henrique, D. and Bekman, E. (2009). Neural differentiation of embryonic stem cells in vitro: a road map to neurogenesis in the embryo. *PLoS One* 4, e6286. 10.1371/journal.pone.0006286 19621087 PMC2709448

[BIO059981C2] Abranches, E., Bekman, E. and Henrique, D. (2013). Generation and characterization of a novel mouse embryonic stem cell line with a dynamic reporter of nanog expression. *PLoS One* 8, e59928. 10.1371/journal.pone.0059928 23527287 PMC3602340

[BIO059981C3] Amel, A., Rossouw, S. and Goolam, M. (2023). Gastruloids: a novel system for disease modelling and drug testing. *Stem Cell Rev. Rep.* 19, 104-113. 10.1007/s12015-022-10462-5 36308705

[BIO059981C4] Antonica, F., Orietti, L. C., Mort, R. L. and Zernicka-Goetz, M. (2019). Concerted cell divisions in embryonic visceral endoderm guide anterior visceral endoderm migration. *Dev. Biol.* 450, 132-140. 10.1016/j.ydbio.2019.03.016 30940540 PMC6553843

[BIO059981C5] Arias, A. M., Marikawa, Y. and Moris, N. (2022). Gastruloids: pluripotent stem cell models of mammalian gastrulation and embryo engineering. *Dev. Biol.* 488, 35-46. 10.1016/j.ydbio.2022.05.002 35537519 PMC9477185

[BIO059981C6] Arkell, R. M. and Tam, P. P. L. (2012). Initiating head development in mouse embryos: integrating signalling and transcriptional activity. *Open Biol.* 2, 120030. 10.1098/rsob.120030 22754658 PMC3382960

[BIO059981C7] Arkell, R. M., Fossat, N. and Tam, P. P. (2013). Wnt signalling in mouse gastrulation and anterior development: new players in the pathway and signal output. *Curr. Opin. Genet. Dev.* 23, 454-460. 10.1016/j.gde.2013.03.001 23608663

[BIO059981C8] Arnold, S. J. and Robertson, E. J. (2009). Making a commitment: cell lineage allocation and axis patterning in the early mouse embryo. *Nat. Rev. Mol. Cell Biol.* 10, 91-103. 10.1038/nrm2618 19129791

[BIO059981C9] Baillie-Johnson, P., Van Den Brink, S. C., Balayo, T., Turner, D. A. and Martinez Arias, A. (2015). Generation of aggregates of mouse embryonic stem cells that show symmetry breaking, polarization and emergent collective behaviour in vitro. *J. Vis. Exp*. 105: 53252. 10.3791/53252 PMC4692741 26650833

[BIO059981C10] Bardot, E. S. and Hadjantonakis, A. K. (2020). Mouse gastrulation: coordination of tissue patterning, specification and diversification of cell fate. *Mech. Dev.* 163, 103617. 10.1016/j.mod.2020.103617 32473204 PMC7534585

[BIO059981C11] Beccari, L., Moris, N., Girgin, M., Turner, D. A., Baillie-Johnson, P., Cossy, A.-C., Lutolf, M. P., Duboule, D. and Arias, A. M. (2018). Multi-axial self-organization properties of mouse embryonic stem cells into gastruloids. *Nature* 562, 272-276. 10.1038/s41586-018-0578-0 30283134

[BIO059981C12] Bedzhov, I. and Zernicka-Goetz, M. (2014). Self-organizing properties of mouse pluripotent cells initiate morphogenesis upon implantation. *Cell* 156, 1032-1044. 10.1016/j.cell.2014.01.023 24529478 PMC3991392

[BIO059981C13] Bedzhov, I., Leung, C. Y., Bialecka, M. and Zernicka-Goetz, M. (2014). In vitro culture of mouse blastocysts beyond the implantation stages. *Nat. Protoc.* 9, 2732-2739. 10.1038/nprot.2014.186 25356584

[BIO059981C14] Berenger-Currias, N. M., Mircea, M., Adegeest, E., Van Den Berg, P. R., Feliksik, M., Hochane, M., Idema, T., Tans, S. J. and Semrau, S. (2022). A gastruloid model of the interaction between embryonic and extra-embryonic cell types. *J. Tissue Eng.* 13, 20417314221103042. 10.1177/20417314221103042 35707767 PMC9189523

[BIO059981C15] Bottenstein, J. E. and Sato, G. H. (1979). Growth of a rat neuroblastoma cell line in serum-free supplemented medium. *Proc. Natl. Acad. Sci. USA* 76, 514-517. 10.1073/pnas.76.1.514 284369 PMC382972

[BIO059981C16] Brewer, G. J., Torricelli, J. R., Evege, E. K. and Price, P. J. (1993). Optimized survival of hippocampal neurons in B27-supplemented Neurobasal, a new serum-free medium combination. *J. Neurosci. Res.* 35, 567-576. 10.1002/jnr.490350513 8377226

[BIO059981C17] Canto-Soler, M. V. and Adler, R. (2006). Optic cup and lens development requires Pax6 expression in the early optic vesicle during a narrow time window. *Dev. Biol.* 294, 119-132. 10.1016/j.ydbio.2006.02.033 16564518

[BIO059981C18] Collinson, J. M., Quinn, J. C., Hill, R. E. and West, J. D. (2003). The roles of Pax6 in the cornea, retina, and olfactory epithelium of the developing mouse embryo. *Dev. Biol.* 255, 303-312. 10.1016/S0012-1606(02)00095-7 12648492

[BIO059981C19] Conlon, F. L., Lyons, K. M., Takaesu, N., Barth, K. S., Kispert, A., Herrmann, B. and Robertson, E. J. (1994). A primary requirement for nodal in the formation and maintenance of the primitive streak in the mouse. *Development* 120, 1919-1928. 10.1242/dev.120.7.1919 7924997

[BIO059981C20] Dunty, W. C., Biris, K. K., Chalamalasetty, R. B., Taketo, M. M., Lewandoski, M. and Yamaguchi, T. P. (2008). Wnt3a/β-catenin signaling controls posterior body development by coordinating mesoderm formation and segmentation. *Development* 135, 85-94. 10.1242/dev.009266 18045842

[BIO059981C21] Girgin, M. U., Broguiere, N., Hoehnel, S., Brandenberg, N., Mercier, B., Arias, A. M. and Lutolf, M. P. (2021). Bioengineered embryoids mimic post-implantation development in vitro. *Nat. Commun.* 12, 5140. 10.1038/s41467-021-25237-8 34446708 PMC8390504

[BIO059981C22] Harrison, S. E., Sozen, B., Christodoulou, N., Kyprianou, C. and Zernicka-Goetz, M. (2017). Assembly of embryonic and extraembryonic stem cells to mimic embryogenesis in vitro. *Science* 356, eaal1810. 10.1126/science.aal1810 28254784

[BIO059981C23] Hashmi, A., Tlili, S., Perrin, P., Lowndes, M., Peradziryi, H., Brickman, J. M., Martínez Arias, A. and Lenne, P.-F. (2022). Cell-state transitions and collective cell movement generate an endoderm-like region in gastruloids. *Elife* 11, e59371. 10.7554/eLife.59371 35404233 PMC9033300

[BIO059981C24] Haushalter, C., Asselin, L., Fraulob, V., Dollé, P. and Rhinn, M. (2017). Retinoic acid controls early neurogenesis in the developing mouse cerebral cortex. *Dev. Biol.* 430, 129-141. 10.1016/j.ydbio.2017.08.006 28790015

[BIO059981C25] Heidari Khoei, H., Javali, A., Kagawa, H., Sommer, T. M., Sestini, G., David, L., Slovakova, J., Novatchkova, M., Scholte Op Reimer, Y. and Rivron, N. (2023). Generating human blastoids modeling blastocyst-stage embryos and implantation. *Nat. Protoc.* 18, 1584-1620. 10.1038/s41596-023-00802-1 36792779 PMC7617227

[BIO059981C26] Hu, X., Gao, J., Liao, Y., Tang, S. and Lu, F. (2013). Retinoic acid alters the proliferation and survival of the epithelium and mesenchyme and suppresses Wnt/β-catenin signaling in developing cleft palate. *Cell Death Dis.* 4, e898. 10.1038/cddis.2013.424 24176856 PMC3920929

[BIO059981C27] Izumi, N., Era, T., Akimaru, H., Yasunaga, M. and Nishikawa, S. (2007). Dissecting the molecular hierarchy for mesendoderm differentiation through a combination of embryonic stem cell culture and RNA interference. *Stem Cells* 25, 1664-1674. 10.1634/stemcells.2006-0681 17446562

[BIO059981C28] Kanai-Azuma, M., Kanai, Y., Gad, J. M., Tajima, Y., Taya, C., Kurohmaru, M., Sanai, Y., Yonekawa, H., Yazaki, K., Tam, P. P. L. et al. (2002). Depletion of definitive gut endoderm in Sox17-null mutant mice. *Development* 129, 2367-2379. 10.1242/dev.129.10.2367 11973269

[BIO059981C29] Keller, P. J. (2013). Imaging morphogenesis: technological advances and biological insights. *Science* 340, 1234168. 10.1126/science.1234168 23744952

[BIO059981C30] Kim, D. H., Xing, T., Yang, Z., Dudek, R., Lu, Q. and Chen, Y. H. (2017). Epithelial mesenchymal transition in embryonic development, tissue repair and cancer: a comprehensive overview. *J. Clin. Med.* 7, 1. 10.3390/jcm7010001 29271928 PMC5791009

[BIO059981C31] Kumar, A., Lualdi, M., Lyozin, G. T., Sharma, P., Loncarek, J., Fu, X. Y. and Kuehn, M. R. (2015). Nodal signaling from the visceral endoderm is required to maintain Nodal gene expression in the epiblast and drive DVE/AVE migration. *Dev. Biol.* 400, 1-9. 10.1016/j.ydbio.2014.12.016 25536399 PMC4806383

[BIO059981C32] Libby, A. R. G., Joy, D. A., Elder, N. H., Bulger, E. A., Krakora, M. Z., Gaylord, E. A., Mendoza-Camacho, F., Butts, J. C. and Mcdevitt, T. C. (2021). Axial elongation of caudalized human organoids mimics aspects of neural tube development. *Development* 148, dev198275. 10.1242/dev.198275 34142711 PMC8254868

[BIO059981C33] Liu, P., Wakamiya, M., Shea, M. J., Albrecht, U., Behringer, R. R. and Bradley, A. (1999). Requirement for Wnt3 in vertebrate axis formation. *Nat. Genet.* 22, 361-365. 10.1038/11932 10431240

[BIO059981C34] Morrisey, E. E., Tang, Z., Sigrist, K., Lu, M. M., Jiang, F., Ip, H. S. and Parmacek, M. S. (1998). GATA6 regulates HNF4 and is required for differentiation of visceral endoderm in the mouse embryo. *Genes Dev.* 12, 3579-3590. 10.1101/gad.12.22.3579 9832509 PMC317242

[BIO059981C35] Nowotschin, S., Hadjantonakis, A.-K. and Campbell, K. (2019). The endoderm: a divergent cell lineage with many commonalities. *Development* 146, dev150920. 10.1242/dev.150920 31160415 PMC6589075

[BIO059981C36] Olmsted, Z. T. and Paluh, J. L. (2022). A combined human gastruloid model of cardiogenesis and neurogenesis. *iScience* 25, 104486. 10.1016/j.isci.2022.104486 35721464 PMC9198845

[BIO059981C37] Osei-Sarfo, K. and Gudas, L. J. (2014). Retinoic acid suppresses the canonical Wnt signaling pathway in embryonic stem cells and activates the noncanonical Wnt signaling pathway. *Stem Cells* 32, 2061-2071. 10.1002/stem.1706 24648413 PMC4106995

[BIO059981C38] Osteil, P., Studdert, J. B., Goh, H. N., Wilkie, E. E., Fan, X., Khoo, P.-L., Peng, G., Salehin, N., Knowles, H., Han, J.-D. J. et al. (2019). Dynamics of Wnt activity on the acquisition of ectoderm potency in epiblast stem cells. *Development* 146, dev172858. 10.1242/dev.172858 30890572

[BIO059981C39] Osumi, N., Shinohara, H., Numayama-Tsuruta, K. and Maekawa, M. (2008). Concise review: Pax6 transcription factor contributes to both embryonic and adult neurogenesis as a multifunctional regulator. *Stem Cells* 26, 1663-1672. 10.1634/stemcells.2007-0884 18467663

[BIO059981C40] Perea-Gomez, A., Lawson, K. A., Rhinn, M., Zakin, L., Brulet, P., Mazan, S. and Ang, S. L. (2001). Otx2 is required for visceral endoderm movement and for the restriction of posterior signals in the epiblast of the mouse embryo. *Development* 128, 753-765. 10.1242/dev.128.5.753 11171400

[BIO059981C41] Perea-Gomez, A., Camus, A., Moreau, A., Grieve, K., Moneron, G., Dubois, A., Cibert, C. and Collignon, J. (2004). Initiation of gastrulation in the mouse embryo is preceded by an apparent shift in the orientation of the anterior-posterior axis. *Curr. Biol.* 14, 197-207. 10.1016/j.cub.2004.01.030 14761651

[BIO059981C42] Peng, G., Suo, S., Chen, J., Chen, W., Liu, C., Yu, F., Wang, R., Chen, S., Sun, N., Cui, G. et al. (2016). Spatial Transcriptome for the Molecular Annotation of Lineage Fates and Cell Identity in Mid-gastrula Mouse Embryo. *Dev. Cell* 36, 681-697. 10.1016/j.devcel.2016.02.020 27003939

[BIO059981C43] Probst, S., Sagar, Tosic, J., Schwan, C., Grün, D. and Arnold, S. J. (2021). Spatiotemporal sequence of mesoderm and endoderm lineage segregation during mouse gastrulation. *Development* 148, dev193789. 10.1242/dev.193789 33199445

[BIO059981C44] Rivera-Perez, J. A. and Magnuson, T. (2005). Primitive streak formation in mice is preceded by localized activation of Brachyury and Wnt3. *Dev. Biol.* 288, 363-371. 10.1016/j.ydbio.2005.09.012 16289026

[BIO059981C45] Rivron, N. C., Frias-Aldeguer, J., Vrij, E. J., Boisset, J.-C., Korving, J., Vivié, J., Truckenmüller, R. K., Van Oudenaarden, A., Van Blitterswijk, C. A. and Geijsen, N. (2018). Blastocyst-like structures generated solely from stem cells. *Nature* 557, 106-111. 10.1038/s41586-018-0051-0 29720634

[BIO059981C46] Ross, S. A., Mccaffery, P. J., Drager, U. C. and De Luca, L. M. (2000). Retinoids in embryonal development. *Physiol. Rev.* 80, 1021-1054. 10.1152/physrev.2000.80.3.1021 10893430

[BIO059981C47] Rossi, G., Broguiere, N., Miyamoto, M., Boni, A., Guiet, R., Girgin, M., Kelly, R. G., Kwon, C. and Lutolf, M. P. (2021). Capturing cardiogenesis in gastruloids. *Cell Stem Cell* 28, 230-240.e6. 10.1016/j.stem.2020.10.013 33176168 PMC7867643

[BIO059981C48] Schrode, N., Saiz, N., Di Talia, S. and Hadjantonakis, A.-K. (2014). GATA6 levels modulate primitive endoderm cell fate choice and timing in the mouse blastocyst. *Dev. Cell* 29, 454-467. 10.1016/j.devcel.2014.04.011 24835466 PMC4103658

[BIO059981C49] Shahbazi, M. N. and Zernicka-Goetz, M. (2018). Deconstructing and reconstructing the mouse and human early embryo. *Nat. Cell Biol.* 20, 878-887. 10.1038/s41556-018-0144-x 30038253

[BIO059981C50] Simunovic, M. and Brivanlou, A. H. (2017). Embryoids, organoids and gastruloids: new approaches to understanding embryogenesis. *Development* 144, 976-985. 10.1242/dev.143529 28292844 PMC5358114

[BIO059981C51] Sozen, B., Amadei, G., Cox, A., Wang, R., Na, E., Czukiewska, S., Chappell, L., Voet, T., Michel, G., Jing, N. et al. (2018). Self-assembly of embryonic and two extra-embryonic stem cell types into gastrulating embryo-like structures. *Nat. Cell Biol.* 20, 979-989. 10.1038/s41556-018-0147-7 30038254

[BIO059981C52] Sozen, B., Cox, A. L., De Jonghe, J., Bao, M., Hollfelder, F., Glover, D. M. and Zernicka-Goetz, M. (2019). Self-organization of mouse stem cells into an extended potential blastoid. *Dev. Cell* 51, 698-712.e8. 10.1016/j.devcel.2019.11.014 31846649 PMC10291877

[BIO059981C53] Tam, P. P. L. and Loebel, D. A. F. (2007). Gene function in mouse embryogenesis: get set for gastrulation. *Nat. Rev. Genet.* 8, 368-381. 10.1038/nrg2084 17387317

[BIO059981C54] Taniguchi, K., Heemskerk, I. and Gumucio, D. L. (2019). Opening the black box: stem cell-based modeling of human post-implantation development. *J. Cell Biol.* 218, 410-421. 10.1083/jcb.201810084 30552099 PMC6363460

[BIO059981C55] Ten Berge, D., Koole, W., Fuerer, C., Fish, M., Eroglu, E. and Nusse, R. (2008). Wnt signaling mediates self-organization and axis formation in embryoid bodies. *Cell Stem Cell* 3, 508-518. 10.1016/j.stem.2008.09.013 18983966 PMC2683270

[BIO059981C56] Teo, A. K., Arnold, S. J., Trotter, M. W., Brown, S., Ang, L. T., Chng, Z., Robertson, E. J., Dunn, N. R. and Vallier, L. (2011). Pluripotency factors regulate definitive endoderm specification through eomesodermin. *Genes Dev.* 25, 238-250. 10.1101/gad.607311 21245162 PMC3034899

[BIO059981C57] Teo, A. K., Valdez, I. A., Dirice, E. and Kulkarni, R. N. (2014). Comparable generation of activin-induced definitive endoderm via additive Wnt or BMP signaling in absence of serum. *Stem Cell Rep.* 3, 5-14. 10.1016/j.stemcr.2014.05.007 PMC4110751 25068117

[BIO059981C58] Thomas, P. and Beddington, R. (1996). Anterior primitive endoderm may be responsible for patterning the anterior neural plate in the mouse embryo. *Curr. Biol.* 6, 1487-1496. 10.1016/S0960-9822(96)00753-1 8939602

[BIO059981C59] Thomson, M., Liu, S. J., Zou, L.-N., Smith, Z., Meissner, A. and Ramanathan, S. (2011). Pluripotency factors in embryonic stem cells regulate differentiation into germ layers. *Cell* 145, 875-889. 10.1016/j.cell.2011.05.017 21663792 PMC5603300

[BIO059981C60] Turner, D. A., Hayward, P. C., Baillie-Johnson, P., Rué, P., Broome, R., Faunes, F. and Martinez Arias, A. (2014a). Wnt/β-catenin and FGF signalling direct the specification and maintenance of a neuromesodermal axial progenitor in ensembles of mouse embryonic stem cells. *Development* 141, 4243-4253. 10.1242/dev.112979 25371361 PMC4302903

[BIO059981C61] Turner, D. A., Trott, J., Hayward, P., Rue, P. and Martinez Arias, A. (2014b). An interplay between extracellular signalling and the dynamics of the exit from pluripotency drives cell fate decisions in mouse ES cells. *Biol. Open* 3, 614-626. 10.1242/bio.20148409 24950969 PMC4154298

[BIO059981C62] Turner, D. A., Baillie-Johnson, P. and Martinez Arias, A. (2016). Organoids and the genetically encoded self-assembly of embryonic stem cells. *BioEssays* 38, 181-191. 10.1002/bies.201500111 26666846 PMC4737349

[BIO059981C63] Turner, D. A., Girgin, M., Alonso-Crisostomo, L., Trivedi, V., Baillie-Johnson, P., Glodowski, C. R., Hayward, P. C., Collignon, J., Gustavsen, C., Serup, P. et al. (2017). Anteroposterior polarity and elongation in the absence of extraembryonic tissues and spatially localised signalling in Gastruloids, mammalian embryonic organoids. *Development* 144, 3894-3906. 10.1242/dev.150391 28951435 PMC5702072

[BIO059981C64] Van Den Brink, S. C. and Van Oudenaarden, A. (2021). 3D gastruloids: a novel frontier in stem cell-based in vitro modeling of mammalian gastrulation. *Trends Cell Biol.* 31, 747-759. 10.1016/j.tcb.2021.06.007 34304959

[BIO059981C65] Van Den Brink, S. C., Baillie-Johnson, P., Balayo, T., Hadjantonakis, A. K., Nowotschin, S., Turner, D. A. and Martinez Arias, A. (2014). Symmetry breaking, germ layer specification and axial organisation in aggregates of mouse embryonic stem cells. *Development* 141, 4231-4242. 10.1242/dev.113001 25371360 PMC4302915

[BIO059981C66] Van Den Brink, S. C., Alemany, A., Van Batenburg, V., Moris, N., Blotenburg, M., Vivié, J., Baillie-Johnson, P., Nichols, J., Sonnen, K. F., Martinez Arias, A. et al. (2020). Single-cell and spatial transcriptomics reveal somitogenesis in gastruloids. *Nature* 582, 405-409. 10.1038/s41586-020-2024-3 32076263

[BIO059981C67] Veenvliet, J. V., Bolondi, A., Kretzmer, H., Haut, L., Scholze-Wittler, M., Schifferl, D., Koch, F., Guignard, L., Kumar, A. S., Pustet, M. et al. (2020). Mouse embryonic stem cells self-organize into trunk-like structures with neural tube and somites. *Science* 370, eaba4937. 10.1126/science.aba4937 33303587

[BIO059981C68] Veenvliet, J. V., Lenne, P. F., Turner, D. A., Nachman, I. and Trivedi, V. (2021). Sculpting with stem cells: how models of embryo development take shape. *Development* 148, dev192914. 10.1242/dev.192914 34908102 PMC8722391

[BIO059981C69] Vincent, S. D., Dunn, N. R., Hayashi, S., Norris, D. P. and Robertson, E. J. (2003). Cell fate decisions within the mouse organizer are governed by graded Nodal signals. *Genes Dev.* 17, 1646-1662. 10.1101/gad.1100503 12842913 PMC196136

[BIO059981C70] Wang, J., Sinha, T. and Wynshaw-Boris, A. (2012). Wnt signaling in mammalian development: lessons from mouse genetics. *Cold Spring Harbor Perspect. Biol.* 4, a007963. 10.1101/cshperspect.a007963 PMC3331704 22550229

[BIO059981C71] Xu, P. F., Borges, R. M., Fillatre, J., De Oliveira-Melo, M., Cheng, T., Thisse, B. and Thisse, C. (2021). Construction of a mammalian embryo model from stem cells organized by a morphogen signalling centre. *Nat. Commun.* 12, 3277. 10.1038/s41467-021-23653-4 34078907 PMC8172561

[BIO059981C72] Yamaguchi, T. P. (2001). Heads or tails: Wnts and anterior-posterior patterning. *Curr. Biol.* 11, R713-R724. 10.1016/S0960-9822(01)00417-1 11553348

[BIO059981C73] Yeung, J., Ha, T. J., Swanson, D. J. and Goldowitz, D. (2016). A Novel and Multivalent Role of Pax6 in Cerebellar Development. *J. Neurosci.* 36, 9057-9069. 10.1523/JNEUROSCI.4385-15.2016 27581449 PMC5005719

[BIO059981C74] Ying, Q.-L., Stavridis, M., Griffiths, D., Li, M. and Smith, A. (2003). Conversion of embryonic stem cells into neuroectodermal precursors in adherent monoculture. *Nat. Biotechnol.* 21, 183-186. 10.1038/nbt780 12524553

[BIO059981C75] Ying, Q.-L., Wray, J., Nichols, J., Batlle-Morera, L., Doble, B., Woodgett, J., Cohen, P. and Smith, A. (2008). The ground state of embryonic stem cell self-renewal. *Nature* 453, 519-523. 10.1038/nature06968 18497825 PMC5328678

[BIO059981C76] Yu, L., Wei, Y., Duan, J., Schmitz, D. A., Sakurai, M., Wang, L., Wang, K., Zhao, S., Hon, G. C. and Wu, J. (2021). Blastocyst-like structures generated from human pluripotent stem cells. *Nature* 591, 620-626. 10.1038/s41586-021-03356-y 33731924

[BIO059981C77] Zhang, S. (2014). Sox2, a key factor in the regulation of pluripotency and neural differentiation. *World J Stem Cells* 6, 305. 10.4252/wjsc.v6.i3.305 25126380 PMC4131272

[BIO059981C78] Zylicz, J. J. (2020). Defined stem cell culture conditions to model mouse blastocyst development. *Curr. Protoc. Stem Cell Biol.* 52, e105. 10.1002/cpsc.105 31971672

