# Supplementary material for: Wnt and BMP signalling direct anterior–posterior differentiation in aggregates of mouse embryonic stem cells
Source: Biol Open. 2023 Sep 13;12(9):bio059981. doi: 10.1242/bio.059981 (PMC10508691; doi:10.1242/bio.059981)
Supplement: Supplementary information [file biolopen-12-059981-s1.pdf]

Table S1. Primer sequences used for qPCR

Gene	Forward sequence (5' to 3')	Reverse sequence (5' to 3')	Amplicon size (bp)
Gapdh	AGGTCGGTGTGAACGGATTTG	TGTAGACCATGTAGTTGAGGTCA	123
Oct4	CAATGCCGTGAAGTTGGAGAAG	GGCTGAACACCTTTCCAAAGAGA	179
Nanog	TCTTCCTGGTCCCCACAGTTT	GCAAGAATAGTTCTCGGGATGAA	100
Sox2	GCGGAGTGGAAACTTTTGTCC	CGGGAAGCGTGTACTTATCCTT	157
Sox17	GATGCGGGATACGCCAGTG	CCACCACCTCGCCTTTCAC	136
Gata6	GTGGTCGCTTGTGTAGAAGGA	TTGCTCCGGTAACAGCAGTG	105
Brachyury	GCTGGATTACATGGTCCCAAG	GGCACTTCAGAAATCGGAGGG	158
Mixl1	GTCTTCCGACAGACCATGTACC	CCCGCCTTGAGGATAAGGG	160
Pou3f1	TTCAAGCAACGACGCATCAA	TGCGAGAACACGTTACCGTAGA	86
Slc7a3	TTCTGGCCGAGTTGTCTATGTTTG	AGTGCGGTTCTGTGGCTGTCTC	190
Otx2	GAATCCAGGGTGCAGGTATGG	CTGAACTCACTTCCCGAGCTG	136
E-cadherin	CAGGTCTCCTCATGGCTTTGC	CTTCCGAAAAGAAGGCTGTCC	175
Nodal	CCTGGAGCGCATTTGGATG	ACTTTTCTGCTCGACTGGACA	155
Snai1	CTTGTGTCTGCACGACCTGT	ACATCCGAGTGGGTTTGGAG	167
Pax6	TACCAGTGTCTACCAGCCAAT	TGCACGAGTATGAGGAGGTCT	194
ß-catenin	ATGGAGCCGGACAGAAAAGC	CTTGCCACTCAGGGAAGGA	108
Eomes	TCGCTGTGACGGCCTACCAA	AGGGGAATCCGTGGGAGATGGA	210
Wnt3	CTCGCTGGCTACCCAATTTG	CTTGACACCTTCTGCTACGCT	165
Bmp4	TGTGAGGAGTTTCCATCACGA	CAGGAACCATTTCTGCTGGGG	230
En1	ACACAACCCTGCGATCCTACT	GGACGGTCCGAATAGCGTG	120
Irx6	TGGTGTCTGCAAGTTCCAGTG	AGCCGACTGTCATAGGGTGT	118

Biology Open • Supplementary information

Table S2. StepOnePlus™ Real-Time PCR run parameters for a 2hr run.

Stage	Step	Temperature (°C)	Duration
Holding	1	95	10 min
Cycling (40 cycles)	1	95	15 s
	2	60	1 min
Melt Curve (continuous)	1	95	15 s
	2	60	1 min
	3	95 (with a ramp rate of 2.8%)	15 s

Biology Open • Supplementary information
